# Pentosan Polysulfate: Oral Versus Subcutaneous Injection in Mucopolysaccharidosis Type I Dogs

**DOI:** 10.1371/journal.pone.0153136

**Published:** 2016-04-11

**Authors:** Calogera M. Simonaro, Shunji Tomatsu, Tracy Sikora, Francyne Kubaski, Michael Frohbergh, Johana M. Guevara, Raymond Y. Wang, Moin Vera, Jennifer L. Kang, Lachlan J. Smith, Edward H. Schuchman, Mark E. Haskins

**Affiliations:** 1 Department of Genetics and Genomic Sciences, Icahn School of Medicine at Mount Sinai, New York, NY, United States of America; 2 Skeletal Dysplasia Laboratory, Nemours/Alfred I. duPont Hospital for Children, Wilmington, DE, United States of America; 3 School of Veterinary Medicine, University of Pennsylvania, Philadelphia, PA, United States of America; 4 Pontificia Universidad Javeriana, Bogota, Colombia; 5 Division of Metabolic Disorders, Children’s Hospital of Orange County, Orange, CA, United States of America; 6 Division of Medical Genetics, Department of Pediatrics, Harbor-UCLA Medical Center, Torrance, CA, United States of America; 7 Department of Neurosurgery, University of Pennsylvania, Philadelphia, PA, United States of America; Baylor Research Institute, UNITED STATES

## Abstract

**Background:**

We previously demonstrated the therapeutic benefits of pentosan polysulfate (PPS) in a rat model of mucopolysaccharidosis (MPS) type VI. Reduction of inflammation, reduction of glycosaminoglycan (GAG) storage, and improvement in the skeletal phenotype were shown. Herein, we evaluate the long-term safety and therapeutic effects of PPS in a large animal model of a different MPS type, MPS I dogs. We focused on the arterial phenotype since this is one of the most consistent and clinically significant features of the model.

**Methodology/Principal Findings:**

MPS I dogs were treated with daily oral or biweekly subcutaneous (subQ) PPS at a human equivalent dose of 1.6 mg/kg for 17 and 12 months, respectively. Safety parameters were assessed at 6 months and at the end of the study. Following treatment, cytokine and GAG levels were determined in fluids and tissues. Assessments of the aorta and carotid arteries also were performed. No drug-related increases in liver enzymes, coagulation factors, or other adverse effects were observed. Significantly reduced IL-8 and TNF-alpha were found in urine and cerebrospinal fluid (CSF). GAG reduction was observed in urine and tissues. Increases in the luminal openings and reduction of the intimal media thickening occurred in the carotids and aortas of PPS-treated animals, along with a reduction of storage vacuoles. These results were correlated with a reduction of GAG storage, reduction of clusterin 1 staining, and improved elastin integrity. No significant changes in the spines of the treated animals were observed.

**Conclusions:**

PPS treatment led to reductions of pro-inflammatory cytokines and GAG storage in urine and tissues of MPS I dogs, which were most evident after subQ administration. SubQ administration also led to significant cytokine reductions in the CSF. Both treatment groups exhibited markedly reduced carotid and aortic inflammation, increased vessel integrity, and improved histopathology. We conclude that PPS may be a safe and useful therapy for MPS I, either as an adjunct or as a stand-alone treatment that reduces inflammation and GAG storage.

## Introduction

The mucopolysaccharidoses (MPS) are group of 11 lysosomal storage disorders (LSDs) due to deficiencies of enzymes that degrade glycosaminoglycans (GAGs) [1,2]. MPS type I results from deficient activity of alpha-L-iduronidase (IDUA), leading to the accumulation of partially degraded heparan and dermatan sulfate fragments in fluids and tissues. It is a clinically heterogeneous disorder and commonly classified into three subtypes: Hurler (MPS IH), Scheie (MPS IS), and Hurler/Scheie (MPS IH/S). GAG accumulation in all MPS types has a direct effect on connective tissue formation and function, resulting in an array of skeletal, skin and other connective tissue abnormalities, as well as lesions in other organs such as the central nervous system (CNS). In addition, GAG accumulation in this and other MPS types activates the toll like receptor 4 (TLR4) signaling pathway [3], initiating chronic inflammation that results in proliferation (e.g., of synovioctyes) and apoptosis (e.g., of chondrocytes) of connective tissue cells, and further exacerbates the disease pathology. [4,5].

Hematopoietic stem cell transplantation (HSCT) and enzyme replacement therapies (ERT) are available for MPS patients, but neither treatment completely ameliorates GAG accumulation or the progression of disease [6,7]. Residual GAG accumulation is particularly prominent in tissues such as the heart, brain, and the musculoskeletal system, which are less accessible to exogenously delivered enzymes [8,9]. Early treatment may partially improve these effects [10,11], although cardiac valve disease, corneal clouding and skeletal changes still do not respond well [12]. For example, while ERT may improve some features of the upper airway disease in MPS patients (e.g., narrowed or blocked nasal passages), other elements, such as trachea degeneration, generally do not improve [13]. ERT also has little or no effect on the CNS. HSCT may similarly slow disease progression in MPS patients provided that the transplant is successful and engraftment of enzyme-expressing donor cells is high, but the effects on the skeletal and neurological systems are limited and the disease continues to progress [6]. Long-term HSCT outcome studies in MPS IH patients have shown considerable residual disease burden, with high variability between patients. Preservation of cognitive function and a younger age at transplantation were major predictors for superior cognitive development post transplant [14]. Thus, there is a clear medical need for new MPS therapies that can be used alone or in conjunction with ERT and/or HSCT, which formed the basis of the current study.

Pentosan polysulfate (PPS) is a sulfated polysaccharide polymer isolated from beech trees [15]. It has potent anti-inflammatory and weak anti-coagulant activity, and has been marketed in Europe for over 30 years with a very positive safety profile. It is also used in veterinary medicine to treat osteoarthritis [16,17]. We have previously demonstrated that PPS treatment in MPS VI rats reduced inflammation and resulted in significant pathological and clinical improvements [18,19]. We also found that PPS reduced GAG storage in the MPS animals, an unexpected result that was confirmed in urine and tissues. The effects of subQ administration (once weekly) were greater than for daily oral treatment in the MPS VI rat model, particularly in avascular tissues such as the cartilage and bone.

The current study was designed to evaluate the effectiveness of PPS in a large animal model of a different MPS type, canine MPS I due to a homozygous null mutation in intron 1 of the *IDUA* gene [20]. MPS I dogs are a model with intermediate severity, similar to the Hurler-Scheie phenotype in humans, [9,21,22]. Herein, we compared the two modes of PPS administration in affected MPS I dogs; daily oral treatment for 17 months and bi-weekly subQ treatment for 12 months. Both groups of animals were started on treatment at 3 weeks of age. The main goal of the study was to establish the safety of chronic PPS treatment in a large animal model of MPS prior to the initiation of clinical trials. Several secondary endpoints also were studied, including effects on GAG storage, inflammation markers, and improvement in the arterial phenotype. The latter was chosen as a clinical endpoint due to its consistent presentation in affected animals and the documented influence of inflammation on the cardiac lesions [9].

## Materials and Methods

### Animals

The MPS I canine model has been previously described and used extensively [21–23]. The dogs, cross-bred mongrels, were bred and maintained at the University of Pennsylvania School of Veterinary Medicine under NIH and USDA guidelines for the care and use of animals in research. The IACUC of the University of Pennsylvania approved all procedures # 804280. Animals had *ad libitum* access to food (standard Teklad canine lab chow) and water, housing with enrichment, and a 12 hour light/dark cycle. Housing consisted of expanded mesh flooring, and involved housing with compatible conspecifics as required for social species unless medically or behaviorally contraindicated. MPS I affected animals of both sexes were identified at birth by DNA mutation analysis. Euthanasia by overdose of sodium barbiturate (80mg/kg IV) in accordance with the American Veterinary Association and NIH guidelines. The weight of the animals at the time of euthanasia was between 12 and 26 kg.

#### Treatment of MPS I Dogs with PPS

Powdered PPS was obtained from bene pharmaChem (Germany) and freshly dissolved in sterile saline before each use. Three week-old MPS I dogs received 1.6 mg/kg of PPS (human equivalent dose, HED) oral every day for 17 months or subQ once every two weeks for 12 months (n = 5/group). Untreated affected littermates were used as controls (n = 5). At 6 months and at the end of treatment (12 and 17 months, respectively) blood was drawn to assess various safety parameters, including general chemistries/K9 panel, K9 CBC, coagulation panel, and liver enzymes. All measurements were performed in the clinical laboratory at the Ryan Veterinary Hospital, University of Pennsylvania, and values were compared to established K9 normal levels. Untreated MPS I dogs of the same age were used as disease controls. Blood was also collected for the quantification of inflammatory cytokines. At the end of the study (i.e., one month after the last PPS dose) the animals were euthanized by overdose of sodium barbiturate (80mg/kg IV) in accordance with the American Veterinary Association and NIH guidelines. Tissues were collected and either frozen or fixed in neutral buffered 10% formalin (Sigma Chemical, St. Louis, MO) for analysis. Urine also was collected for GAG determinations (see below).

### Cytokine Determinations

Inflammatory cytokines were assessed in serum and cerebrospinal fluid (CSF) by enzyme-linked immunosorbent assays (ELISAs) using canine ELISA kits according to the manufacturers’ protocols. For canine TNF-α and interleukin-8 (IL-8) # CAT00 and CA8000 from R & D Systems (Minneapolis, MN) were used, respectively. All assays were performed in triplicate.

### Total GAG Determinations

Tissues (aorta, liver, spleen, kidney cortex, and kidney medulla) were homogenized and total GAGs determined according to the BLYSCAN GAG kit #NC0287381 protocol (Fisher Scientific, Walthman, MA). Total urine GAGs were measured using the same kit followed by creatinine assays using the Microvue creatinine assay kit #NC9732564 (Fisher Scientific, Walthman, MA).

### Mass Spectometry GAG Analysis

10μl of urine was added to a 96 well omega 10K filter plate (Pall Co, MI) with 90 μl of 50mM Tris HCL (pH 7.0) with a 96-well receiver plate at the bottom. Samples were incubated for 15 min and then centrifuged for 15 min at 2200g. After centrifugation, the 96-well bottom plate was replaced for a new plate and the samples were incubated with a mixture of: 60 μl of 50mM Tris HCL (pH 7.0), 10 μl of 5 μg/ml of internal standard (IS, Chondrosine), 10 μl of 1 mU chondroitinase B (in BSA 1%), 10 μl of 1 mU heparitinase (in BSA 1%), and 10 μl of 1 mU keratanase II (in BSA 1%) (enzymes and IS were provided by Seikagaku Co, Tokyo). Samples were incubated overnight in a 37°C water bath, followed by a centrifugation for 15 min at 2200 rcf and then injected to the LC-MS/MS. The LC-MS/MS was a 1260 Infinity Degasser, 1290 infinity thermostat, 6460 triple quad mass spectrometer (Agilent Technologies, CA). The method used was based on the method described by Oguma et al. [23]. Specific precursor ion and *m/z* were used to quantify each disaccharide, respectively (354.3, 193.1 IS; 462, 97 mono-KS; 542, 462 di-KS; 416, 138 ΔDiHS-NS; 378.3, 175.1 ΔDiHS-0S; 458, 175 ΔDiHS-6S; 458.4–300.2 DS). The injection volume was 5 μl with a running time of 4.5 min per sample.

### Histopathology

Descending aortas and left and right carotids were collected from normal, untreated and PPS-treated MPS I dogs. The tissues were either frozen or fixed with 4% paraformaldehyde/PBS. The fixed tissues were paraffin-embedded and 6 μm sections were prepared. Slides were stained either with H&E or Van Gieson stain for elastin (Sigma Aldrich, kit# Ht25A). H&E sections were used to quantify the dimensions of the vessels.

For immunohistochemistry, the paraffin-sections were permeabilized with 0.5% Triton-X-100, blocked, and incubated overnight at 4°C with anti-clusterin antibody (#05–354, Millipore, Temecula, CA). The Lab Vision Ultravision Detection System (Thermo Scientific, US) was used according to the manufacturer’s instructions and mounted with an anti-bleaching mounting media. Slides were visualized and photographed with a confocal laser-scanning microscope (Carl Zeiss 510 Meta).

### Cervical Spine Analyses

Prior to euthanasia, magnetic resonance images (MRI) were obtained of the cervical spine from normal, untreated MPS I dogs, and MPS I dogs treated with oral PPS for 12 months. MRI imaging was not performed on MPS I dogs treated with subQ PPS. MR images were obtained using a 1.5T Signa LX magnet (GE Healthcare; Little Chalfont, United Kingdom). Detailed imaging methods were published previously [24]. Disc degenerative condition also was assessed on these animals using the semi-quantitative Pfirrmann grading scheme [25], where scores range from 1 (healthy disc) to 5 (severely degenerate), and spinal cord compression severity was semi-quantitatively assessed using the scheme of Dickson et al [26,27]. Disc condition and spinal cord compression severity were calculated for each of 5 cervical spine levels (C2-C7), which were then averaged to produce single mean values for each animal and used for subsequent statistical comparisons.

Following euthanasia, the cervical spine was removed from the animal, the C2 vertebra were isolated, cleaned of surrounding tissue, and scanned using microCT (VivaCT40; Scanco Medical AG, Brüttisellen, Switzerland) by previously published methods [24,27]. MicroCT was performed on all treated animals, both oral and subQ. Standard 3D morphometric analyses of the trabecular bone were performed using Scanco software to calculate bone volume fraction (BV/TV), trabecular thickness (Tb.Th), trabecular spacing (Tb.Sp), trabecular number (Tb.N), and apparent mineral density (bone mineral density, BMD). The entire odontoid process was imaged at the same resolution for qualitative morphological examination.

Finally, C2 vertebral bodies were fixed in 10% neutral buffered formalin for one week, then decalcified using formic acid/EDTA (Formical 2000; Decal Chemical Corporation, Tallman, USA). A 3 mm-thick mid-sagittal slab from each sample was isolated and processed for paraffin-embedded histology. Sections 10 μm thick were double-stained with Alcian blue and picrosirius red to demonstrate GAG and collagen, respectively, and then imaged under bright field light microscopy (Eclipse 90i; Nikon, Tokyo, Japan). The presence or absence of remnant growth plate cartilage in the caudal adjacent to the caudal vertebral end plate was noted.

### Immunoblot Analysis

Healthy, untreated MPS I affected and PPS treated aortas from age-matched dogs were homogenized and pelleted by centrifugation and lysed for immunoblot analysis as previously described [3]. The membranes containing the lysates were incubated with anti-clusterin (#05–354, Millipore, Temecula, CA) and anti-beta-actin (Santa Cruz, CA, #sc-1615, used as a loading control). The bound antibodies were recognized by secondary antibodies conjugated to HRP (GE Healthcare). Detection of the antibody complexes was accomplished using an enhanced chemiluminescence detection reagent (Amersham Biosciences).

### Statistics

A student’s t-test was used to compare values between two groups, and ANOVA with Tukey post hoc analysis was used to compare values between three groups. All statistical analysis was performed using the Sigma Stat software (Systat Software, Inc., Point Richmond, CA).

## Results

### Safety Profiles

MPS I dogs were treated for 17 months with daily oral PPS and biweekly for 12 months by subQ injection beginning at 3 weeks of age (n = 5 per group; 1.6 mg/kg HED). No adverse reactions were reported in either treatment group. Routine blood chemistry and coagulation evaluations were performed, including measuring liver enzymes, at 6 months and at 1 month after stopping treatment, prior to euthanasia (end of study). Table 1 shows values in PPS-treated and untreated MPS I animals at the end of the study. No abnormal changes were observed regardless of the mode of administration.

**Table 1:** Safety Profiles of Oral and Subcutaneous PPS Treatment in MPS I Dogs.

	Creatinine	Total Bilirubin	ALT	AST	ALP	PT	PTT	Platelets
Normal Ranges	0.7–1.8	0.1–0.5	16–91	25–65	20–155	6.8–10.2	10.7–16.4	177–398
Untreated	0.71	0.19	29.37	28.17	77.48	8.04	12.90	439.83*
Oral 17M	0.58	0.20	24.41	22.28	77.0	8.40	13.1	321.00
SQ 12M	0.74	0.10	29.21	23.23	43.8	8.52	13.02	365.54

N = 5 per group; untreated, oral PPS and subcutaneous PPS (SQ)

* = Value is elevated above normal range.

### PPS Reduces Inflammatory Markers in the Serum and CSF of MPS I Dogs

**Fig 1:**
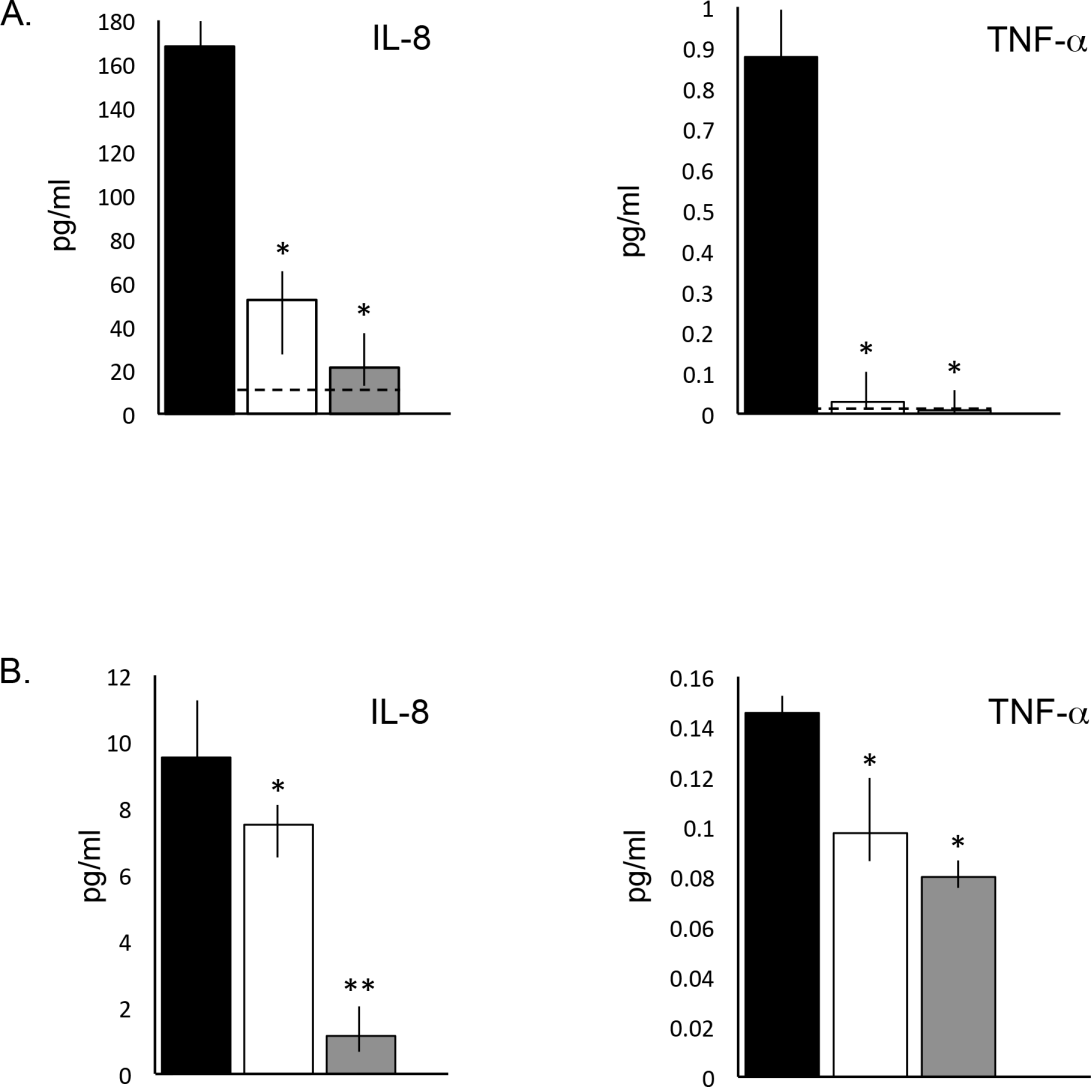
Serum and CSF inflammatory markers in age-matched untreated and PPS-treated MPS I dogs. IL-8 and TNF-alpha were quantified at the end of the PPS treatment (17 months with daily oral treatment and 12 months with biweekly subQ) by ELISA assays as described in the Materials and Methods. (A) Serum IL-8 and TNF-alpha in untreated (black), oral (white), and subQ (grey) groups. * = p<0.005 when comparing treated to untreated animals. Dashed lines represent normal levels. (B) CSF IL-8 and TNF-alpha in the treated and untreated groups. * = p<0.03; ** = p<0.005. The vertical lines in each column indicate the ranges for the individual animals with each group (n = 5/group).

One month after the last PPS administration the treated animals were euthanized and serum, urine, CSF, and tissues were collected. IL-8 and TNF-alpha were measured in the serum and CSF (Fig 1). Significant reductions of both cytokines were observed with both treatment modalities, although greater reductions were obtained from subQ administration, particularly with regard to IL-8 in the CSF. In the serum, PPS was effective in normalizing the levels of TNF-alpha. These results confirmed previous studies in MPS VI rats [18,19] showing reduction of several serum inflammatory cytokines, and reveal for the first time the potential of PPS to influence CNS manifestations.

### GAG Analysis in PPS Treated MPS I Dogs

**Fig 2:**
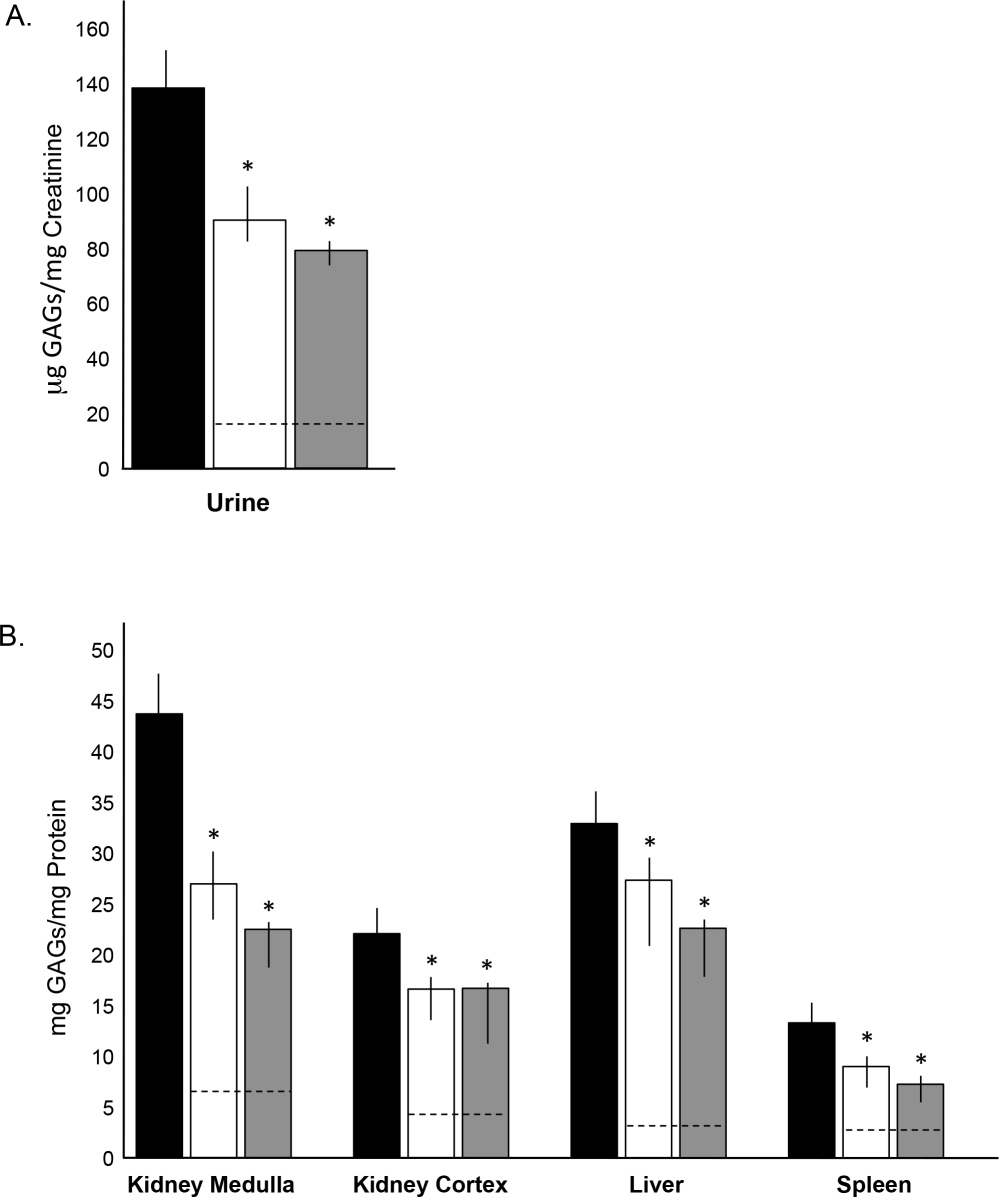
Analysis of total GAGs in age-matched untreated and PPS-treated MPS I dogs. Total GAGs were measured in urine and tissue homogenates at the end of the treatment (17 months with daily oral and 12 months with biweekly subQ PPS. (A) Total urine GAGs were significantly reduced with both modes of PPS administration, with subQ treatment being more significant; * = p<0.02 comparing untreated to oral and * = p<0.01 comparing untreated to subQ. (B) Total GAGs in tissue extracts from the kidney (medulla and cortex), liver and spleen of untreated and PPS-treated animals. In all tissues, PPS significantly lowered GAG storage; as in urine the reductions were greater using subQ administration. * = p<0.05. Black columns, untreated MPS I dogs; white columns, oral PPS treated MPS I dogs; grey columns, subQ PPS-treated MPS I dogs. The vertical lines in each column indicate the ranges for the individual animals in each treatment group (n = 5/group). Dashed lines represent normal levels.

**Fig 3:**
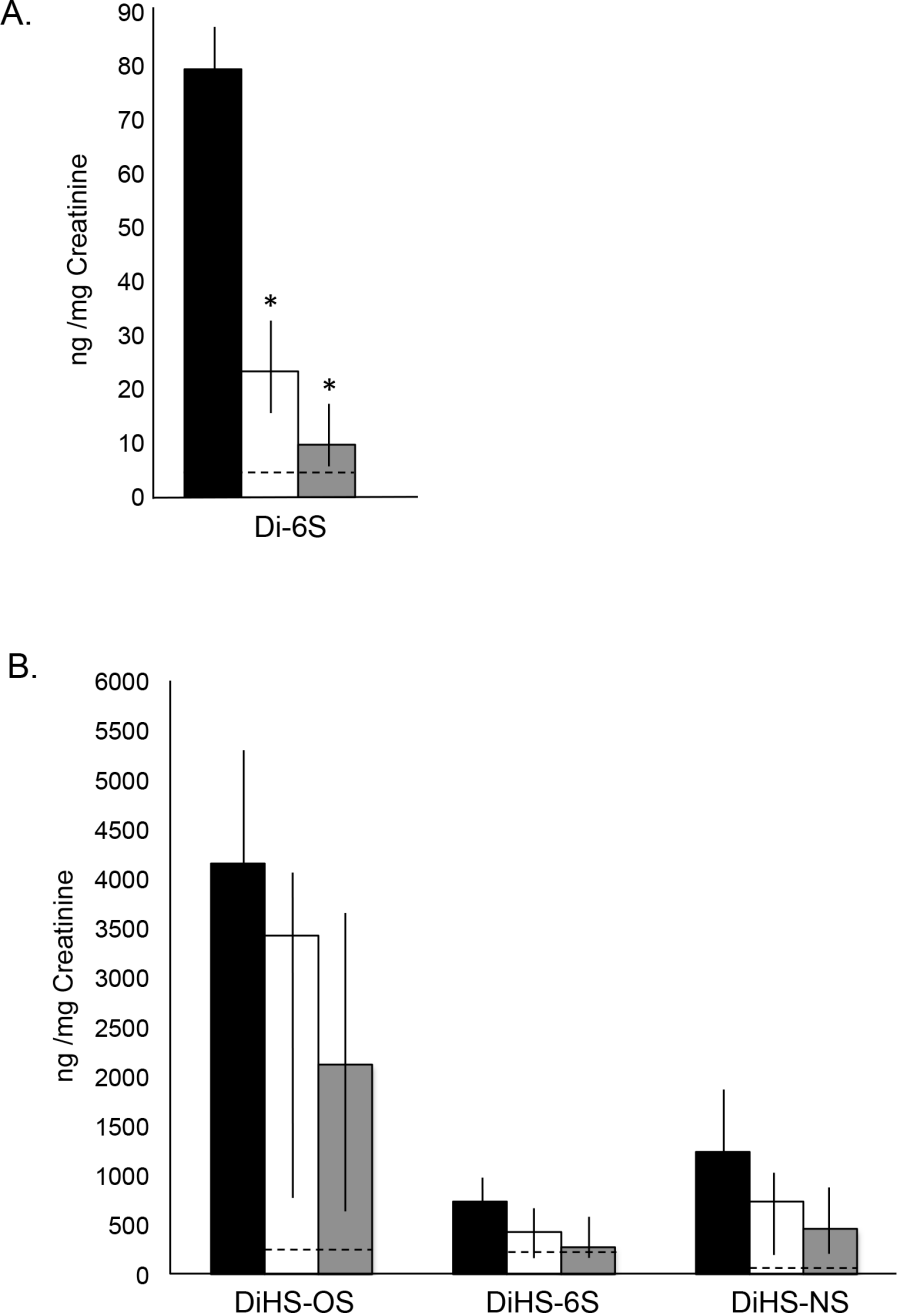
Mass spectrometric analysis of urine GAGs in age-matched normal, untreated and PPS-treated MPS I dogs. Mass spectrometric quantification of GAG-derived disaccharides was performed as described in the Material and Methods. Urine GAGs were measured at the end of the treatment; 17 months with oral daily PPS and 12 months with biweekly subQ PPS. Black columns, untreated MPS I dogs; white columns, oral PPS-treated MPS I dogs; grey columns, subQ PPS-treated MPS I dogs. The vertical lines in each column indicate the ranges. Dashed lines represent normal levels. (A) The dermatan sulfate disaccharide (Di-6S) was significantly reduced with both modes of PPS administration, with subQ treatment being more significant. * p = 0.0232 comparing untreated to oral and p = 0.0141 comparing untreated to subQ. (B) Heparan sulfate disaccharides (DiHS-OS, DiHS-6S, and DiHS-NS) also were reduced with PPS treatment when compared to untreated animals, although the reductions were not significant.

Total GAGs were measured in urine and tissues of age-matched normal, untreated and PPS treated MPS I dogs. Total GAGs were significantly reduced in all treated animals when compared to untreated, with greater reductions seen in the animals treated by subQ PPS (Fig 2). To confirm these findings, mass spectrometry was performed (Fig 3). Heparan sulfate disaccharides (DiHS-OS, DiHS-6S and DiHS-NS) and the dermatan sulfate disaccharide (Di-6S) were each reduced in the PPS treated animals. The greatest response was seen with Di-6S, a primary GAG fragment that also accumulates in MPS VI.

### Effects of PPS on Vascular Lesions

**Fig 4:**
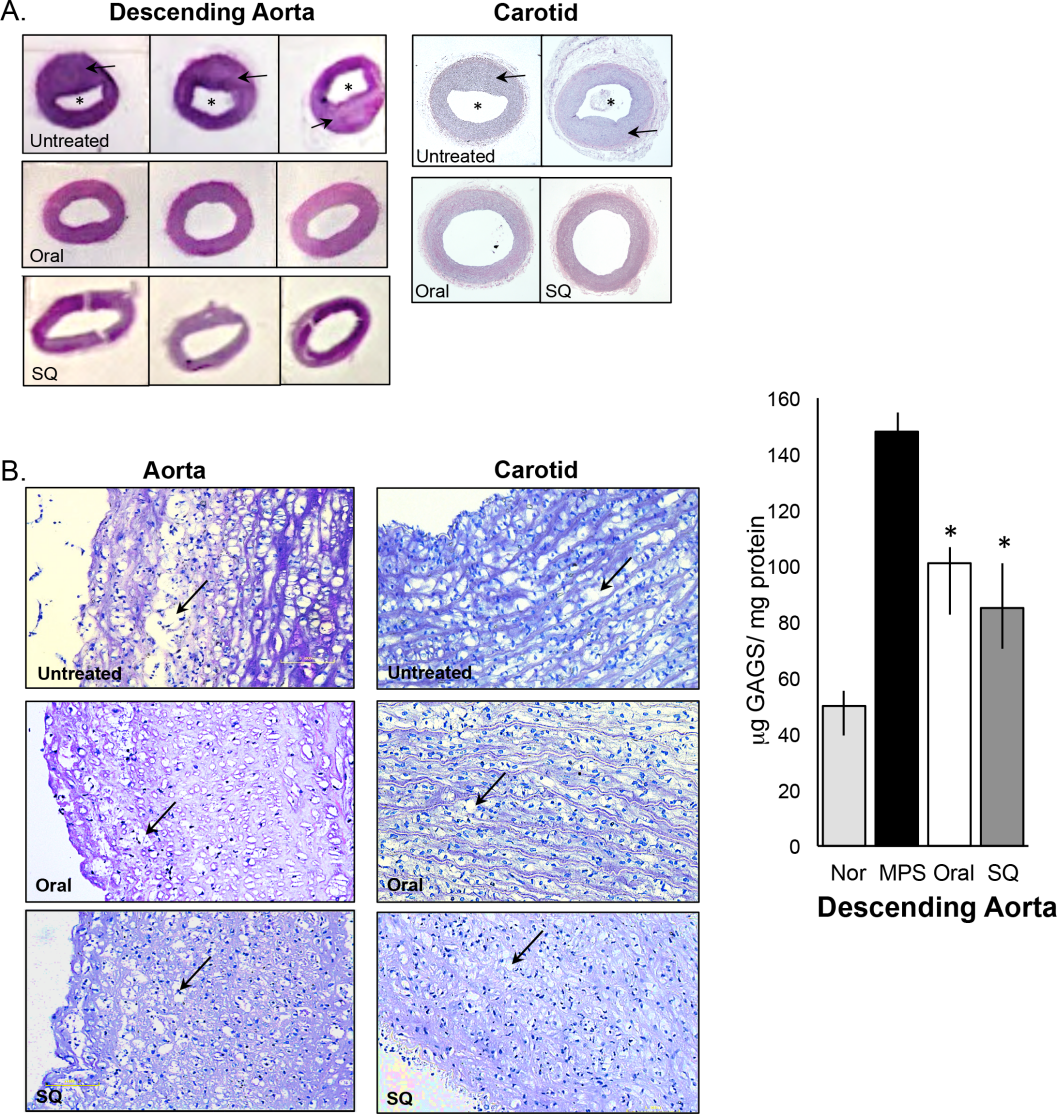
Analysis of the descending aorta and carotid in untreated and PPS-treated MPS I dogs. Representative images (H&E staining) are shown for untreated, oral, and subQ PPS-treated MPS I dogs (17 months with daily oral treatment and 12 months with biweekly subQ). (A) The intima media of both the descending aorta and carotid is thickened (arrows) in the untreated MPS I dog due to lysosomal storage, resulting in narrowing of the lumen (*) (magnification 1X). PPS treatment decreased the thickness of the intimal media and increased the diameter of the lumen with both modes of administration, but was more significant with subQ treatment (see Tables 2 and 3) (B) Higher magnification (10X) of the descending aorta and carotid artery in untreated and PPS-treated MPS I dogs revealed reduced vacuolization/storage in the treated animals when compared to untreated (arrows). (C) Total GAGs were determined in the descending aorta of normal, untreated and PPS-treated MPS I dogs. PPS treatment significantly reduced GAG storage with both modes of treatment, consistent with the histological evidence of storage reduction. Black columns, untreated MPS I dogs; white columns, oral PPS-treated MPS I dogs; grey columns, subQ PPS-treated MPS I dogs. The vertical lines in each column indicate the ranges. * p = 0.0041

Vascular lesions are one the most consistent and clinically relevant features of the MPS I dog model. Yano and Lyons et al. [8,9] have detailed the morphology and potential pathological mechanisms contributing to the vascular lesions in the aortas of MPS I dogs, revealing occlusion of the vascular lumen with asymmetrical, atherosclerotic-like plaques resulting in intima-media thickness. Similar findings were observed in the current study (Fig 4A). Compared to these control animals, age-matched, PPS- treated MPS I dogs had significantly thinner intima and wider lumens. High magnification of the intima (Fig 4B) showed extensive storage vacuoles in the untreated dogs, and decreased vacuolation in the PPS-treated animals (normal aorta S1 Fig.). This was likely due to a reduction in total GAG storage (Fig 4C). Quantitative analysis of the lumen and intimal thickness of the aortas and carotids in normal, PPS treated and untreated MPS I dogs is shown in Tables 2 and 3.

**Table 2:** Quantification of the Lumen and Intimal Thickness of the Aorta In Normal, Untreated and PPS Treated MPS I Dogs.

Aorta	Lumen (μm)	Thickness (μm)
Normal	141.1 (140.1–143.1)	142.7 (140.5–147.1)
Untreated	66.7 (63.6–74.8)	184.6 (174.2–191.9)
Oral PPS	116.4* (110.1–129.5)	144.8* (141.7–170.8)
SQ PPS	141.3*^#^ (137.5–146.9)	155.1*^#^ (145.9–170.9)

*P<0.01 when comparing untreated to PPS treated animals. There is also a significant effect when comparing oral to SQ treatment

^#^P<0.01. Ranges shown in ().

**Table 3:** Quantification of the Lumen and Intimal Thickness of the Carotid In Normal, Untreated and PPS Treated MPS I Dogs.

	Left	Carotid	Right	Carotid
	Lumen (μm)	Thickness (μm)	Lumen (μm)	Thickness (μm)
Normal	72.5 (69.9–75.9)	73.1 (70.2–77.3)	91.9 (84.2–94.6)	79.9 (77.5–87.1)
Untreated	33.3 (29.9–37.7)	146.7 (108.8–192.7)	61.8 (54.3–64.1)	110.0 (101.5–137.9)
Oral PPS	75.1*(70.1–78.1)	91.8* (86.7–95.9)	85.3* (79.7–89.1)	91.1* (89.1–93.2)
SQ PPS	80.3*^#^ (74.6–80.1)	79.0*^#^(73.7–92.4)	90.1*^#^ (87.4–93.9)	83.8*^#^ (79.9–89.9)

*P<0.01 when comparing untreated to PPS treated animals. There is also a significant effect when comparing oral to SQ treatment

^#^P<0.01. Ranges shown in ().

**Fig 5:**
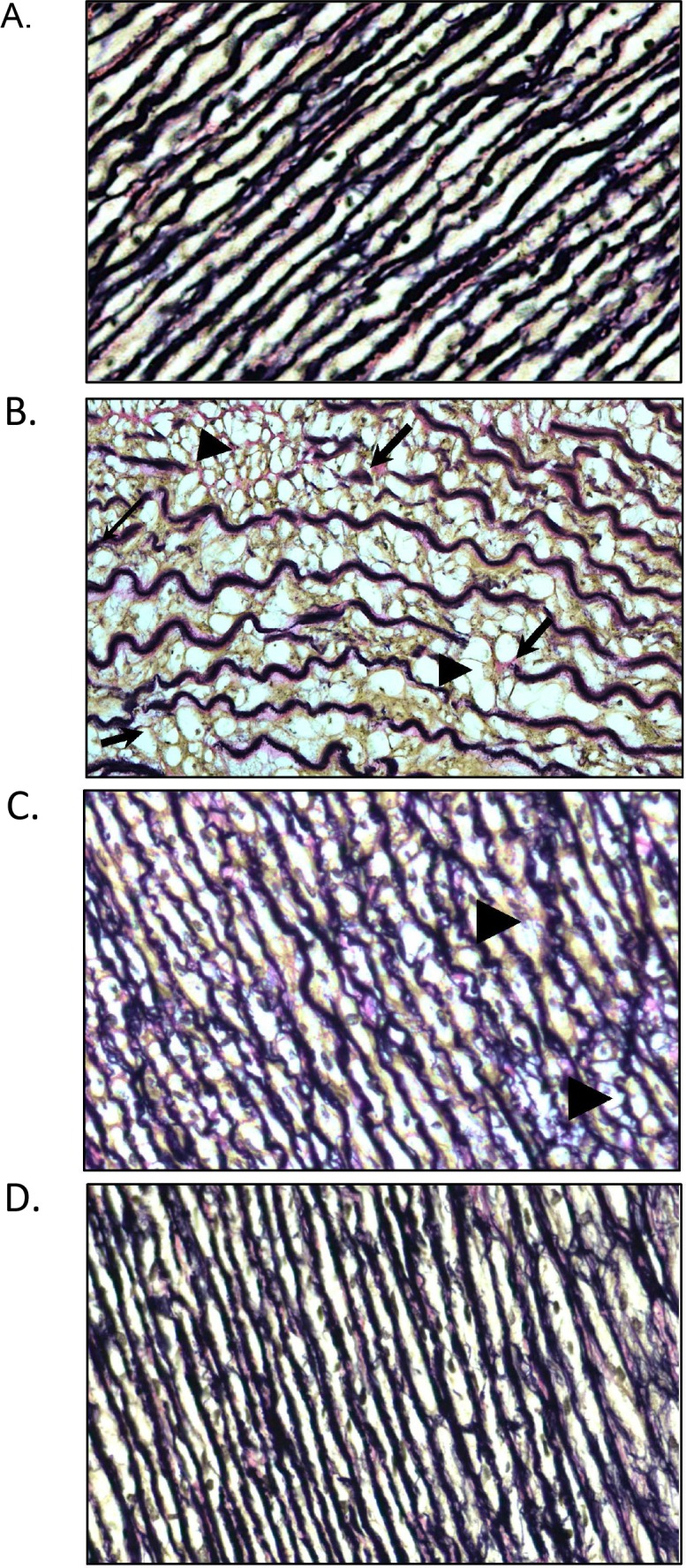
Elastin staining in the descending aorta of normal, untreated and PPS-treated MPS I dogs. (A) Normal (B) Storage (arrowhead) and elastin fragmentation (arrow) is evident in the aortas of untreated 12-month-old MPS I dogs (B) Reduced storage (arrowheads) and fragmentation was observed in MPS I animals treated with oral PPS (daily) for 17 months. (C) Little or no storage or fragmentation was evident in the subQ treated animals (biweekly for 12 months). (20X magnification).

It is also known that the plaques and vacuoles forming in MPS I dogs disrupt the internal elastic lamina of the vessels [9]. This was similarly observed in the untreated MPS I dogs studied here, where extensive elastin fragmentation was observed (Fig 5A). PPS-treated aortas showed almost intact elastin fibers without fragmentation in both the oral and subQ PPS-treated animals (Fig 5B and 5C).

**Fig 6:**
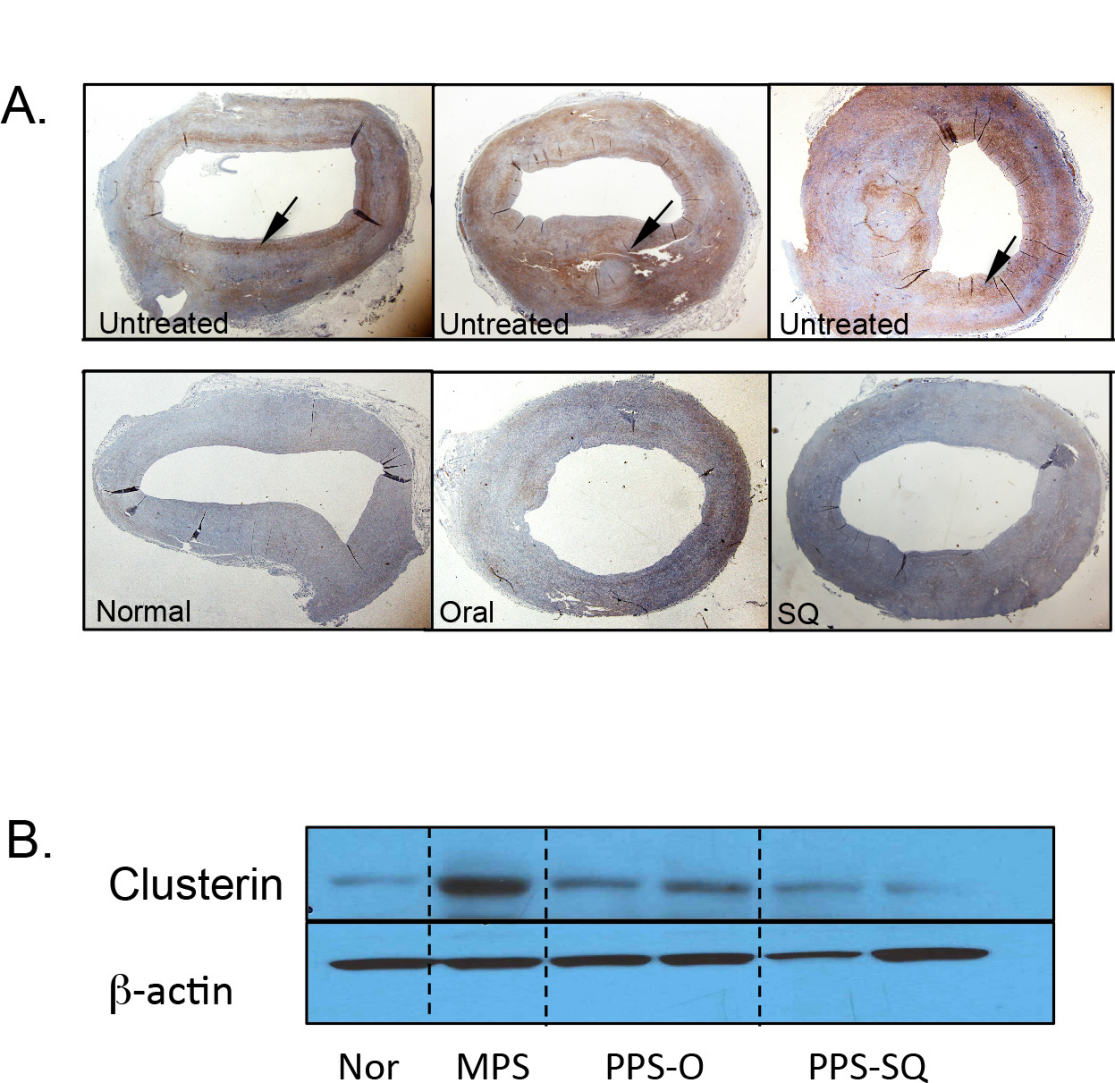
Clusterin expression in the descending aorta of normal, untreated and PPS-treated MPS I dogs. (A) Immunohistochemical analysis of clusterin, a marker of inflammation, in the descending aorta of untreated MPS I dogs. High-level expression (brown) of clusterin in aortic plaques and the inner and outer borders of the vessel were observed in the untreated dogs (arrows). PPS-treated animals (17 months with daily oral and 12 months with biweekly subQ) did not show plaque formation and exhibited reduced clusterin staining similar to normal. Magnification 2X. (B) Western blot analysis showing reduced clusterin expression in the descending aorta of PPS-treated animals. Two representative animals are shown for each treatment group. Nor = age-matched normal animal, PPS-O = MPS I dogs treated with daily oral PPS for 17 months. PPS-SQ = MPS dogs treated with biweekly subQ with PPS for 12 months.

Lyons et al. [9] also have demonstrated activation of the NFKB signaling pathway in the MPS I plaques. Khalid et al. [28] have found that clusterin (*CLU*, also known as apolipoprotein J) was 9-fold overexpressed in treatment-naive MPS I canine aorta compared to normal canine aorta. The clusterin protein is of particular interest as a potential biomarker of MPS I cardiovascular disease because it is involved in morphologic transformation of vascular smooth muscle cells [29] and present in human atherosclerotic plaques, but not in healthy normal aortas [30]. Therefore, we examined clusterin in both the carotids and aortas of the PPS-treated animals using immunohistochemistry and immunoblots (Fig 6). The areas surrounding the plaques and the outer edges of the intima showed increased clusterin expression in the untreated vessels (Fig 6A), while PPS-treated animals exhibited reduced staining similar to normal. Reduced expression of clusterin following PPS treatment also was confirmed by Western blot analysis, with almost complete normalization observed in the subQ treated group (Fig 6B).

### Effects of PPS on the Cervical Spine

Intervertebral disc condition, spinal cord compression, and vertebral body trabecular bone properties in the cervical spines of PPS-treated MPS I dogs were examined using MRI, microCT, and histological analyses and compared to age-matched normal and untreated MPS I dogs. No MRI changes or improvements in disc condition or spinal cord compression were noted in the MPS I dogs treated by oral PPS for 17 months (data not shown). Comparative MRI analysis was not performed on the subQ treated dogs. MicroCT analysis revealed modest, but not significant, improvements in BV/TV, BMD, and Tb/Th in the subQ treated animals only (S2 Fig). Histological analysis revealed some evidence of reduced storage, more evident in the subQ animals, although this could not be quantified (S3 Fig)

## Discussion

It is widely recognized that the existing enzyme and stem cell therapies for MPS patients do not completely ameliorate the disease symptoms [6,7]. Progressive skeletal, cardiac, neurological, and other disease manifestations continue to progress, even when the therapies are initiated early in life. Our research has therefore focused on understanding the mechanism(s) underlying the skeletal phenotype in MPS, with the long-term goal of developing new therapies that can either be adjuncts to existing treatments or stand-alone therapies [3,19,20]. An optimal therapy for MPS should be safe, cost effective, easy to administer, and act against a mechanism common to all MPS types.

PPS was originally developed in Germany as an anti-coagulant for the treatment of deep vein thrombosis [31]. It has been used in Germany for over 30 years as an injected drug (SP54^®^), and as an oral formulation (Elmiron^®^) in the United States to treat Interstitial Cystitis [32,33]. Thousands of patients have been treated with PPS and the safety profile is well documented. Although PPS has weak anti-coagulant properties compared to heparin and other more commonly used anti-thrombotic drugs, subsequent research revealed potent anti-inflammatory properties [34–37]. The anti-inflammatory mechanism of PPS remains unknown, although research has suggested that direct binding of various pro-inflammatory molecules may be responsible [38].

We have previously documented the important role of the TLR4/TNF-alpha pathway in MPS skeletal pathology, leading us to evaluate PPS in a rat model of MPS VI [18, 39]. Reduction of inflammation and improvement in skeletal and other clinical features were documented. Weekly subQ administration of PPS led to better effects than daily oral treatment, presumably due to the greater bioavailability of the subQ delivered drug compared to oral (~15% absorption compared to <2%, respectively, *unpublished data from bene pharmaChem*). Of particular surprise was that GAG reduction was observed in tissues of the MPS VI rats, particularly following subQ treatment. The mechanism of how PPS prevents or reduces GAG storage in MPS is currently under investigation, with hypotheses ranging from inhibition of new GAG synthesis, chaperone effects on the mutant enzyme(s), and/or improvement of lysosomal integrity.

Since we have shown that different GAGs activate the TLR4 pathway to a similar degree, we hypothesized that PPS should be effective in other MPS types, leading us to the current study that compared oral vs. subQ PPS treatment in MPS I dogs [3]. The canine model of MPS I has been used extensively to develop ERT for MPS I patients [40]. It has a moderate phenotype that most resembles MPS IH/S, with slowly progressing skeletal and neurological features. One of the most consistent and clinically relevant aspects of the model is the arterial phenotype, where affected dogs present with arterial plaque formation, inflammation, and other structural abnormalities [9]. The animals also survive over 2 years, allowing long-term treatment and evaluation. This is important since chronic PPS injections have not been used in any human patient population, including MPS, and the safety needs to be determined. Treatment of the MPS VI rats was performed for up to 6 months without any deleterious effects.

The dose used in this dog study (1.6 mg/kg) was based on previous MPS VI rat studies showing optimal effects at 2 mg/kg, and is within the safe dose range used for SP54^®^. All animal doses were adjusted to human equivalent doses (HEDs) according to standard conversion charts [41]. Oral administration was performed daily, while subQ was given once every other week. MPS I animals were started on treatment at 3 weeks of age, and the study was designed to evaluate the: a) safety of chronic PPS treatment over time, and b) prevention of the arterial phenotype. Urine and serum cytokine and GAG assays also were performed. The study design called for animals to be treated for at least one year and for monthly blood samples to be drawn to measure various safety parameters, including thrombotic molecules and liver enzymes. Animals receiving oral treatment were extended to 17 months. At the end of the study all of the animals were euthanized along with age-matched, untreated MPS I control dogs, and the tissues and fluids were analyzed.

Most important, no adverse reaction to either PPS treatment was observed, and at no time during the study was any elevation of safety markers observed. As expected from the MPS VI rat results, both PPS treatments prevented the elevation of TNF-alpha and IL-8 in the serum. Of surprise, however, PPS also prevented elevation of these cytokines in the CSF, particularly IL-8. SubQ administration was more effective in the CSF, presumably due to the greater bioavailability. There is little literature examining whether PPS is able to cross the blood brain barrier, however, direct injection of PPS into the CNS of patients with Creutzfeld-Jakob disease resulted in reduction of inflammation and improvement in brain pathology in a small number of patients [42–44]. It is unclear how PPS reduces cytokines in the CSF of MPS I dogs, and the brains of these animals were not collected since untreated MPS I dogs of this age do not develop clinically significant brain disease. However, these results suggest that PPS should be systematically evaluated in other neurological MPS models (e.g., MPS III) in the future to further assess the ability of the drug to influence brain disease, alone or in conjunction with other CNS-directed therapies.

Another important aspect of this study was the effect of PPS on the prevention of GAG storage, confirming the observations described in MPS VI rats [19]. Results were confirmed by analysis of total GAGs and by mass spectrometric analysis of GAG-specific disaccharides. While both heparan- and dermatan sulfate-related disaccharides were reduced by PPS, the effects on the dermatan sulfate fragments were somewhat greater. This is consistent with the clinical effects of PPS in the MPS VI rats, a model in which dermatan sulfate is the major accumulating GAG. In the future it will be important to evaluate PPS in animal models of other MPS types with different patterns of GAG storage to better understand which MPS would be most amenable to PPS treatment.

The other primary aim of this study was to evaluate the effects of PPS on the arterial phenotype. Both PPS treatments prevented inflammation and plaque formation in the aortas and carotid arteries of the MPS I dogs, although the effects of subQ administration were somewhat greater than oral. This was particularly evident from the immunoblots of clusterin and by GAG analysis, again attributed to the better bioavailability of the subQ drug. Arterial disease is an important feature of MPS that is not particularly well treated by existing ERTs, and this result could have important clinical implications for patients.

This study also assessed changes in the spines of the PPS-treated MPS I dogs. Cervical spinal disease is an important and debilitating feature of MPS, and has been assessed previously in this canine model [27]. For example, MicroCT analysis of vertebrae from MPS I dogs at 3, 6 and 12 months of age showed lower trabecular bone volume/total volume (BV/TV), trabecular thickness (Tb.Th), trabecular number (Tb.N) and bone mineral density (BMD) than normal dogs at all ages. The odontoid process also appeared morphologically abnormal in MPS I dogs at 6 and 12 months of age, and affected animals had significantly more cartilage in the vertebral epiphyses. In addition, the epiphyseal growth plate was absent in 12 month old normal dogs whereas in the MPS I dogs they persisted. In the current study MRI analysis was only carried out on dogs treated with oral PPS due to funding constraints, and did not reveal any drug related improvements. MicroCT analysis was performed on both sets of treated animals and revealed modest improvements in some parameters (BV/TV, BMD, and Tb.Th), but only in the subQ treated animals. Reduced storage also was observed in the vertebral bodies of both treatment groups, more evident in the subQ group. These studies suggest that some modest improvements may be achievable by PPS treatment in MPS spines, although they likely will require subQ administration for at least 2 years.

Overall, we conclude that PPS should continue to be investigated for the treatment of MPS, particularly as a subQ formulation that may be given biweekly or perhaps less frequently. There are several ways to envision PPS use for MPS; either as an adjunct together with ERTs or as a stand-alone therapy. The latter may be particularly amenable in the context of newborn screening to treat MPS children prior to the initiation of ERTs, or used in patients who cannot receive ERT either because it is not available or because of immune responses. Lastly, it is important to note that based on these animal studies two small proof-of-concept clinical trials have recently been concluded in adult MPS I and II patients receiving ERTs (unpublished results). The drug was administered subQ for up to 6 months and no drug-related adverse events were observed. Several positive trends were observed in urine GAG reductions and clinical improvements.

## Supporting Information

S1 Fig Normal 12 month-old canine aorta showing large lumen and thin walls (2x) and no storage at higher magnification (10X). (TIF)

S2 Fig MicroCT analysis of the cervical spines from untreated and PPS treated MPS I dogs. (TIF)

S3 Fig Histological analysis of the vertebral bodies from age-matched normal control, untreated and PPS treated MPS I dogs. (TIF)
