# Supplementary figures and images for: Pentosan Polysulfate: Oral Versus Subcutaneous Injection in Mucopolysaccharidosis Type I Dogs
Source: PLoS One. 2016 Apr 11;11(4):e0153136. doi: 10.1371/journal.pone.0153136 (PMC4827827; doi:10.1371/journal.pone.0153136)

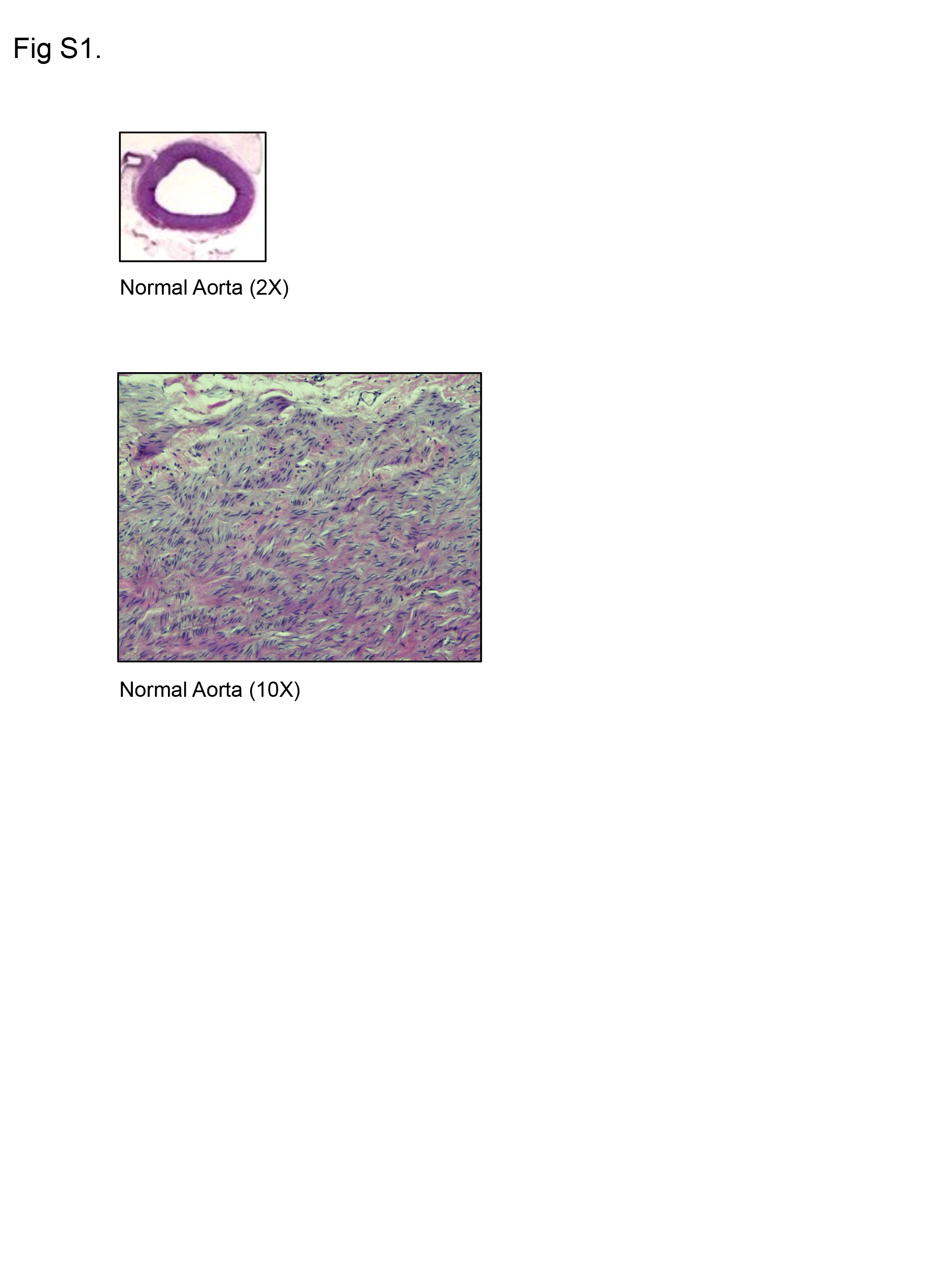

Supplement: S1 Fig — (TIF) [file pone.0153136.s001.tif]

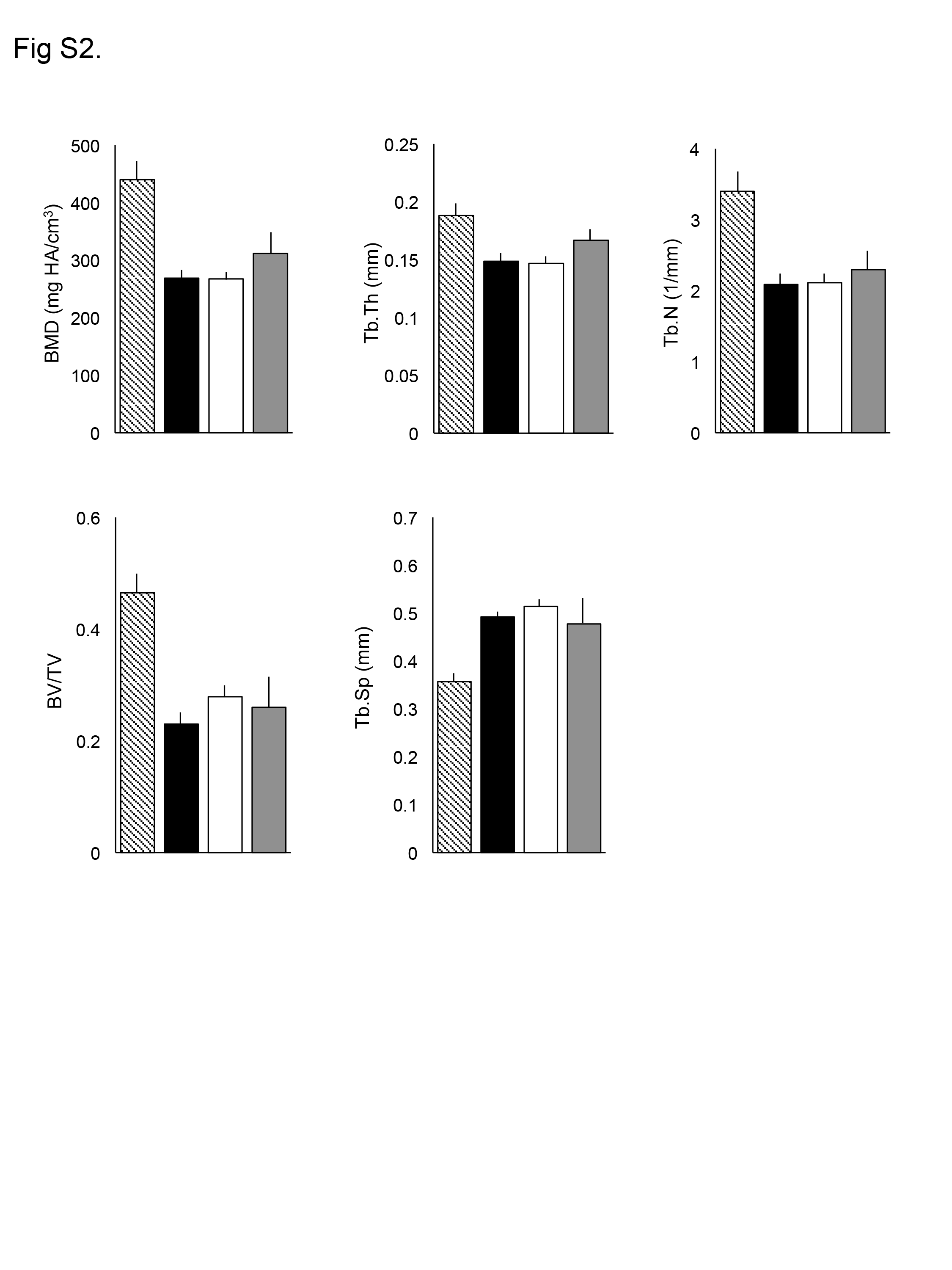

Supplement: S2 Fig — (TIF) [file pone.0153136.s002.tif]

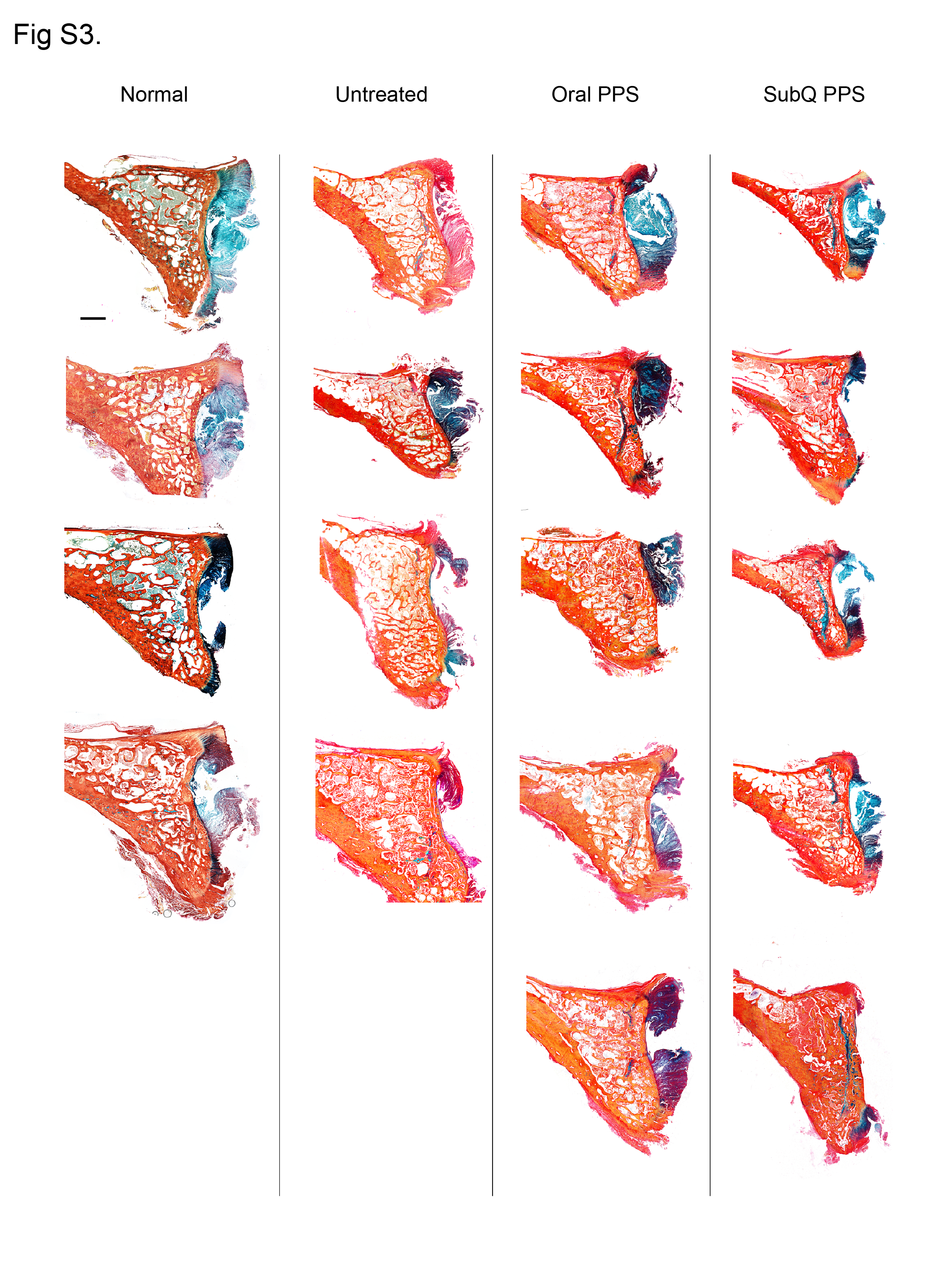

Supplement: S3 Fig — (TIF) [file pone.0153136.s003.tif]
